# Draft Genome Sequence of *Falsirhodobacter* sp. Strain alg1, an Alginate-Degrading Bacterium Isolated from Fermented Brown Algae

**DOI:** 10.1128/genomeA.00826-14

**Published:** 2014-08-21

**Authors:** Tetsushi Mori, Mami Takahashi, Reiji Tanaka, Toshiyuki Shibata, Kouichi Kuroda, Mitsuyoshi Ueda, Haruko Takeyama

**Affiliations:** a Faculty of Science and Engineering, Waseda University Center for Advanced Biomedical Sciences, Shinju-ku, Tokyo, Japan; b Department of Life Sciences, Graduate School of Bioresources, Mie University, Tsu, Mie, Japan; c Division of Applied Life Sciences, Graduate School of Agriculture, Kyoto University, Kitashirakawa Oiwake-cho, Sakyo-ku, Kyoto, Japan; d JST-CREST, Chiyoda-ku, Tokyo, Japan

## Abstract

*Falsirhodobacter* sp. alg1 is an alginate-degrading bacterium, the second species from the nonphototrophic bacterial genus *Falsirhodobacter*. We report the first draft genome of a bacterium from this genus and point out possible important features related to alginate assimilation and its evolutionary aspects.

## GENOME ANNOUNCEMENT

Alginate-degrading bacteria are considered important targets since they harbor significant genes for the assimilation of alginate, a major component for the production of bioethanol from brown algae (1, 2). These bacteria are also important for evolutionary studies to understand the as yet unclear mechanisms behind alginate assimilation (3). Here, we introduce the draft genome sequence of a novel alginate-degrading bacterium, *Falsirhodobacter* sp. alg1 (AB916500), isolated from the supernatant of fermented brown macroalgae, *Eisenia bicyclis*, collected off the shore of Arasaki, Kanagawa Prefecture, Japan. We classified this bacterium in the *Falsirhodobacter* genus (4), because although it showed 97% similarity to bacteria from the phototrophic *Rhodobacter* genus (5), this bacterium also showed nonphototrophic characteristics and had a similarity of 96.6% to the recently reported *Falsirhodobacter halotolerans* (4).

The genome of this bacterium was sequenced by shotgun sequencing using the GS Junior benchtop system (Roche Diagnostics). Two sequence runs were conducted on the bacterial genome, extracted on separate occasions, and the sequence reads attained from both runs were 205,121 bp (average read length, 483 bp) and 170,198 bp (average read length, 413 bp). All sequencing reads from both runs were pooled and *de novo* assembled using the Geneious R7 v. 7.1.2 (Biomatters) (6) to reveal a total of 2,952,029 bp with an average GC content of 60.2%, consisting of 205 contigs (*N*_50_, 413,630 bp). Two of the contigs represented circular plasmids sized at 177,174 bp (FRA_CON006) and 10,356 bp (FRA_CON010). Automatic gene annotation was performed by Rapid Annotation using Subsytem Technology (RAST) (7), and an overview of the annotated genome was viewed using the SEED viewer (8). The genome sequence contains 3,096 coding sequences (CDS), in which 1,599 CDS (52%) were classified into 385 subsystems, while 1,497 (48%) were uncategorized. In addition, 49 tRNA genes for 18 amino acids and 11 rRNA genes (small subunit [SSU], 3; large subunit [LSU], 5; and 5S, 3) were identified.

Analyzing the pathways within the draft genome, we determined the genes related to alginate degradation. Two CDS were shown to encode an alginate lyase precursor (algFR1) (located on FRA_CON002) and an oligonucleotide alginate lyase (algFR2) (located on FRA_CON003), each classified as members of the CAZy (9) alginate lyase families PL-7 and PL-15, respectively. Functional protein analysis by thin-layer chromatography and liquid chromatography-mass spectrometry (LC-MS) showed that both of these alginate lyases have exolytic properties, suggesting that this bacterium has the ability to efficiently degrade alginate to its monomeric unsaturated sugar, 4-deoxy-l-erythro-5-hexoseulose uronate. Subsequently, the search for alginolytic clusters was conducted using the FGENESB online prediction tool (Softberry) (10). Interestingly, we found that algFR2 formed a small operon with several ABC transporter-related genes and an acetoin reductase. In addition, several candidate CDS distributed throughout the genome were also identified that showed similarity to genes found within the alginolytic clusters of other alginate-degrading bacterial strains (3, 11). We believe that *Falsirhodobacter* sp. alg1 may serve as an important target for future research in alginate degradation and evolutionary studies.

### Nucleotide sequence accession numbers.

This whole-genome shotgun project has been deposited at DDBJ/EMBL/GenBank under the accession number BBJC00000000. The version described in this paper is the first version, BBJC01000000.
